# Maternal perceptions of partner support during breastfeeding

**DOI:** 10.1186/1746-4358-8-4

**Published:** 2013-05-08

**Authors:** Cynthia A Mannion, Amy J Hobbs, Sheila W McDonald, Suzanne C Tough

**Affiliations:** 1 University of Calgary, 2500 University Drive NW, Calgary, AB T2N 1N4, Canada; 2 Child Development Centre, c/o 2888 Shaganappi Trail NW, Calgary, Alberta, T3B 6A8, Canada

**Keywords:** Breastfeeding, Perceived support, Partner

## Abstract

**Background:**

Many women find breastfeeding challenging to sustain beyond the first three postpartum months. Women rely on a variety of resources to aid and encourage breastfeeding, including ‘partner support’. Women’s perception of partner support during breastfeeding may influence maternal satisfaction and confidence but it remains understudied. We asked women about their perceptions of partner support during breastfeeding and measured the effect on maternal confidence, commitment, and satisfaction with respect to breastfeeding.

**Methods:**

Using a descriptive, cross sectional design, we recruited 76 mothers from community health clinics in Calgary, Alberta. Participants completed a questionnaire addressing perceptions of partner support, the Breastfeeding Self-Efficacy Scale (BSES) measuring maternal confidence and ability to breastfeed, and the Hill and Humenick Lactation Scale (HHLS) measuring commitment, perceived infant satiety, and breastfeeding satisfaction. Descriptive analysis was performed on socio-demographic and survey responses. Multiple regression modeling was used to examine the association between partner support and breastfeeding outcomes.

**Results:**

Women who reported active/positive support from their partners scored higher on the BSES (p < 0.019) than those reporting ambivalent/negative partner support when we controlled for previous breastfeeding experience and age of infant. There were no significant differences between the two groups of women on total score of HHLS or any of the subscales with respect to perceptions of partner support.

**Conclusion:**

Mothers feel more capable and confident about breastfeeding when they perceive their partners are supportive by way of verbal encouragement and active involvement in breastfeeding activities. Mothers with partners who seemed ambivalent, motivated only by “what’s best for baby,” or provided negative feedback about breastfeeding, felt less confident in their ability to breastfeed. It is important that health care professionals appreciate the influence that positive and active partner support has upon the development of maternal confidence in breastfeeding, a known predictor for maintaining breastfeeding. Common support strategies could be communicated to both the partner and mother in the prenatal and postpartum periods. Health professionals can provide information, invite partners to become active learners and discuss supportive partner functions. Further research should address those functions that are perceived as most supportive by mothers and that partners are willing to perform.

## Background

Efforts to increase breastfeeding rates have been directed at all stages of a woman’s reproductive experience; during the preconception and prenatal periods, within 24 hours of delivery, and throughout the postpartum period in the hospital and at home. Since 1991 the Baby Friendly Hospital Initiative has been implemented in many hospitals and recommends: reduced use of infant formula; nurse assisted initiation of breastfeeding immediately after delivery; the hiring of lactation consultants for post-delivery assistance; and referrals to outside breastfeeding resources upon discharge [1]. None of these efforts seem to increase the duration of breastfeeding.

Breastfeeding duration is highly variable and falls well below the World Health Organization (WHO) and The United Nations Children’s Fund (UNICEF) recommendations of exclusive breastfeeding (no artificial milk substitutes or other fluids) for all infants to six months of age [2]. Most Canadian women try to breastfeed their infants and since 2003 breastfeeding initiation rates in Canada have remained stable at about 87% [3]. However, in 2007, only 23% of mothers reported exclusive breastfeeding for six months [4]. Twenty-five percent of women reported “not enough milk” as the most common reason for stopping breastfeeding. ‘Insufficient milk syndrome’ was recognized in the early 1980s and has persisted to current day as the primary reason women discontinue breastfeeding [5-7]. Although health care professionals offer timely support to breastfeeding women, the more constant presence and immediate support of the baby’s father, or mother’s partner offers opportunity to influence the maintenance and duration of breastfeeding.

Fathers and partners have been identified as being influential in mothers’ feeding decisions and the continuation of breastfeeding [8,9]. If the mother feels that the father’s attitude towards breastfeeding is positive and supportive there is a greater likelihood that she will continue breastfeeding [10,11]. Maternal perception of a negative attitude from their partner influences when a woman considers and decides to discontinue breastfeeding [11].

As part of a larger study looking at breastfeeding self-efficacy and medications used to increase milk supply [12], we asked women about their decisions to breastfeed; to identify breastfeeding supports and to describe their perceptions of their partners’ support and attitudes throughout their breastfeeding experience. It was hypothesized that those mothers reporting positive support from their partners would have higher confidence in breast milk production and higher breastfeeding self-efficacy.

## Methods

### Study design

This study is a descriptive, cross-sectional design using a convenience sample of postpartum mothers. We measured maternal perception of partner’s attitudes towards breastfeeding. The study was conducted in 2009 over a five-month period at six Community Health Centres in the Calgary region where women and breastfeeding infants attended well-baby clinics. Calgary is located in Southern Alberta with a growing and diverse population of approximately one million people. The drop-in clinics operated twice a week in the afternoons until the pandemic H1N1 protocols were initiated and the clinics deployed for vaccination.

### Recruitment

Recruitment posters describing the study were placed in the Community Health Centres. Currently or recently breastfeeding women were approached by clinic staff nurses and referred to research assistants stationed at each clinic. Study participants signed the consent form and completed the demographic questionnaire, the Breastfeeding Self-Efficacy Scale (BSES) and Hill and Humenick Lactation Scale (HHLS) at the clinics. Recruitment occurred over five months period from June 2009 to October 2009 but ceased prematurely as clinics focused on administration of H1N1 vaccinations and mothers and infants were advised to stay away.

Criteria for inclusion were that the study participants had to be English speaking, a mother of a breastfed child, currently with a partner and residing in the Calgary area. Participants were currently breastfeeding or had recently attempted to breastfeed a singleton infant. Exclusion criteria included mothers who had a previous breast reduction or augmentation, illnesses such as breast cancer that required mastectomy or breast lump biopsy, and those who did not have a telephone. Babies born less than 37 weeks gestation and infants with issues that would have complicated breastfeeding, such as a cleft lip or palate, were also excluded.

### Measures

We collected data on variables known to affect breastfeeding. Variables included: type of delivery; current breastfeeding status; previous breastfeeding experience; preparation for breastfeeding; period of time breastfeeding and if formula was used at hospital or at home. Perceived partner support was addressed on the demographic questionnaire with the open-ended questions “Do you feel supported by your partner to breastfeed – why or why not?” and “How do you think your partner feels about breastfeeding?” We also asked women to identify all breastfeeding supports used and when they decided to breastfeed.

The Breastfeeding Self-Efficacy Scale (BSES) is the most widely used instrument employing the self-efficacy concept [13-18]. It is a direct measure of a mother’s confidence in her ability to breastfeed [13]. Several studies have shown that women who have increased confidence in their ability to breastfeed were more likely to continue breastfeeding [14,19]. The BSES has been used extensively for 10 years among a wide age range of breastfeeding women in a variety of populations and has been translated into four languages [20]. Dennis and Faux reported a Cronbach’s alpha co-efficient of 0.96 with 73% of all corrected item-total correlations ranging from 0.3 to 0.70 [14]. The short form BSES is a 14-item self-report instrument where all items are preceded by the phrase “I can always” and anchored with a 5-point Likert scale where 1 = not at all confident and 5 = very confident [14]. Items are summed to produce a score ranging from 14–70 with higher scores indicating higher levels of breastfeeding self-efficacy [14].

### The HHLS and perceived insufficient milk supply

The conceptual framework of perceived insufficient milk supply was used to guide the development of the HHLS [21]. Perceived insufficient milk persists as the primary reason women discontinue breastfeeding [5,6,13,22-24]. The HHLS is a direct measure of the perception women have of their milk production [21]. It has three subscales: maternal commitment, maternal satisfaction, and infant satisfaction. The HHLS is a 20-item self-report instrument where all items are anchored with a 7-point Likert scale where 1 = strongly disagree and 7 = strongly agree and can be used for subscale analysis [21]. Items are summed to produce a score ranging from 20 to 140 with higher scores indicating higher levels of commitment and perceived infant satiety [21]. All subscales show moderate to high internal consistency (alphas 0.75 to 0.98) and concurrent and predictive validity [21]. It has been used with diverse populations [20,25,26].

The study was approved by the Conjoint Health Research Ethics Board of the University of Calgary (E ID-22477).

### Data analysis

Data were entered into a data file using Predictive Analysis Software, (PAWS) 19.0 for analysis. Descriptive statistics (means, standard deviation (SD), frequencies, and percentages) were used to characterize the sample and describe study variables. Chi-square tests and independent sample t-tests were used to explore the relationship between categorical and continuous variables, respectively. Simple and multiple linear regression analyses were used to examine the association between partner support and breastfeeding scales in unadjusted and adjusted models for partner support, adjusting for previous breastfeeding experience and infant age. A p-value of < 0.05 was set as the level of significance.

Participants’ responses to “How do you think your partner feels about breastfeeding?” were collated and categorized by three independent reviewers, as positive, negative or ambivalent. The responses to “Do you feel supported by your partner to breastfeed?” were noted and then matched to the previous question’s categorization for each woman. Where partners exhibited active functions such as “helped position the baby” or “did the housework” women responded they felt supported. We categorized that as positive/active. Comments such as “Glad it's me and not him” and “Does not question me about it” did not elicit feelings of support and we categorized them as negative. Ambivalent comments included “Wants me to do what I’m comfortable with” and “Not sure, I think he would rather me bottle feed”. We collapsed negative and ambivalent answers together for comparison to the active/positive category. The responses from the two questions were re-coded into new variables labeled active/positive support and ambivalent/negative support.

## Results

Seventy-six mothers were recruited to the study and returned questionnaires. The participants were located across the Calgary metropolitan area and represented a range of incomes from all quadrants: the northwest, northeast, southwest, and southeast. One third (n = 25) of the women in our sample were less than 20 weeks postpartum when they joined the study. The mean age of the participants was 31 years (Table 1). The majority of the women in our sample were highly educated and married or living with their partner. One third of women decided to breastfeed during pregnancy (33%, n = 26). Only four women (5%) reported discussing the decision with their partner. Over half (58%, n = 44) reported they were “always going to breastfeed.”

**Table 1:** Demographic, pregnancy and postpartum characteristics for total sample and divided by maternal perception of partner support

**Characteristic**	**Full sample n = 76**	**Active/positive support n = 38**^**a**^	**Ambivalent/negative n = 32**^**a**^	**p- value**^**b**^
More than high school education, n (%)	62 (82)	33 (87)	25 (78)	0.34
Married/living with partner n (%)	74 (97)	36 (95)	32 (100)	0.50 c
Age, mean (SD)	31.2 (4)	31.2 (4)	32.3 (4)	0.22
Infant’s age (weeks)	31 (17)	30.4 (17)	30.4 (17)	0.96
Ever attended prenatal classes, n (%)	58 (76)	29 (76)	24 (75)	0.90
C-section delivery, n (%)	24 (32)	11 (29)	11 (34)	0.67
Self-reported complications with delivery, n (%)	25 (33)	13 (34)	11 (34)	0.99
Previously breastfed a child, n (%)	32 (42)	13 (34)	15 (47)	0.28
Breastfeeding at study contact, (no formula) n (%)	43 (57)	26 (68)	14 (44)	0.04
Did not receive formula in hospital, n (%)	44 (58)	22 (58)	18 (56)	0.89
BSES score by type of support		59.71 SD = 9.33	55.13 SD = 7.58	0.03

^a^Does not add to 76 because 6 women did not report. ^b^ p value of < .0.05 was considered significant. ^c^Fisher’s exact test due to small cell sizes.

Many of the mothers in our study reported that they had attended prenatal classes (76%, n = 56). Fifty-two women (68%) gave birth vaginally and 24 (n = 32%) had surgical deliveries. One third (n = 25) of our sample reported complications with their delivery. Previous breastfeeding experience was reported by 42% (n = 32) of women. Over half the women (58%, n = 44) did not use formula in hospital.

After partner support, the top three breastfeeding supports reported by women were maternal mother (65%, n = 49), friends (65%, n = 49), and physicians (61%, n = 46). Women felt supported by health care professionals at the hospital (92%, n = 70) and following discharge (96%, n = 73) with 77% (n = 59) seeking assistance for breastfeeding post-discharge. Valuable information was received from lactation consultants, nurses and family members. The most useful information women received for breastfeeding came from prenatal classes and health care professionals. The information addressed baby position and latch (25%, n = 15), supportive interaction (29%, n = 17), and advice on how to be patient and to persevere (17%, n = 10) through breastfeeding challenges.

Two categories for perceived maternal support emerged from the analysis of the open ended comments: ‘active/positive’ and ‘ambivalent/negative.’ Active/positive support (n = 38) was characterized by reports of emotional and functional support such as “he is the transporter, brings the baby to me” and “he is very encouraging.” Fifty five per cent of women perceived that their partner was “encouraging,” 23% said their partners thought breastfeeding was best or healthiest for the baby. However, 22% indicated that their partner felt indifferent or negatively about breastfeeding. Ambivalent/negative support (n = 32) was characterized by baby only related comments such as “Does not question me about it” or “good for baby and for the economy.” Frank negative comments about breastfeeding included it was “too time consuming,” “(he) feels that it is healthy for baby but it interferes with intimacy,” and that “(he) feels left out.”

Descriptive characteristics for all women stratified by type of partner support (active/positive and ambivalent/negative) are shown in Table 1. There was a significant difference between the two groups divided by perceived type of support at study contact (p < 0.04). Women who perceived active/positive support from their partners in breastfeeding had higher mean scores of self-efficacy as measured by the BSES than women who reported ambivalent/negative support (Score 59.7 (SD = 9.33) vs. 55.1 (SD = 7.58); p = 0.03). BSES scores ranged from 34 to 70 (total attainable score = 70), while for HHLS the range was 62–115 (total attainable score = 140). There were no significant differences between the two types of perceived support when comparing total HHLS scores or for any of the HHLS subscales. In multiple regression analysis, the effect of active/positive support remained a significant predictor (p = 0.019) of BSES score, controlling for previous breastfeeding experience and age of infant (Table 2).

**Table 2:** Unadjusted and adjusted regression models examining the association between partner support and Breastfeeding Self-efficacy Scale (BSES) and Hill & Humenick Lactation Scale (HHLS) scores

	**BSES**				**HHLS**			
**Independent variable**	**Unadjusted model**		**Adjusted model**^**a**^		**Unadjusted model**		**Adjusted model**^**a**^	
	**β (se)**^**b**^	**p- value**^**c**^	**β (se)**	**p- value**	**β (se)**	**p- value**	**β (se)**	**p- value**
Active positive support	4.59 (2.06)	0.029	4.72 (1.97)	0.019	1.91 (1.92)	0.32	1.98 (1.94)	0.31
Previous breastfeeding experience			1.39 (2.01)	0.49			0.67 (1.97)	0.74
Age of infant (wks)			0.18 (0.06)	0.003			0.07 (0.06)	0.21

^a^Partner support adjusted for previous breastfeeding experience and child age. β(se)^b^ = regression coefficient; se = standard error. ^c^ p < 0.05. dReference group: ambivalent /passive.

## Discussion

In this study we demonstrated that partner support and encouragement were associated with maternal confidence and a perceived ability to breastfeed. Women who experienced positive and active support by their partners showed higher confidence in their ability to breastfeed than women with partners who were ambivalent or negative towards breastfeeding. Partner support was predictive of maternal confidence in breastfeeding regardless of any previous breastfeeding experience or the age of the infant.

In a mixed methods study examining fathers acting as breastfeeding allies, Pontes, Osorio and Alexandrino reported paternal attitudes towards breastfeeding including ambivalence, conflict, exclusion, and insecurity. The effect of these attitudes on maternal breastfeeding was not mentioned [27]. We found similar behaviors perceived by mothers that we termed ambivalent; “(he) is passive about it - goes along with my choices and what I wanted to try;” negative such as “inconvenienced” that adversely affected breastfeeding confidence. Mothers also perceived that partners felt left out and that breastfeeding interfered with intimacy. The effect of breastfeeding on intimacy is seldom mentioned in studies but could be a marker of stress in the re-establishment of sexual activity following childbirth and a topic for sensitive discussion between parents and with a health care professional.

We found that women breastfeeding at the time of the study reported positive perceived paternal support when compared to women who were not breastfeeding and who recalled ambivalent or negative support. Fathers may not recognize how influential active support is to instilling and sustaining the confidence mothers develop in breastfeeding. Active participation characterized by fathers assisting with “domestic chores, and presence during breastfeeding” were perceived by mothers as positive support - “he would sponge my breast before feeding, with warm water” and “he is the ‘transporter’ . . . brings the baby to me for feeding” and “he checked the (baby’s) latch.”

In contrast to earlier findings [10,11,28,29], our study participants did not report that partners were instrumental in their decision to breastfeed. The majority of our sample were “always going to breastfeed,” often considered a predominant factor in infant feeding decisions. A study by Datta, Graham and Wellings found that although fathers were willing to support a mother’s decision to breastfeed, they did not feel that they were able to be a part of the decision making process [30]. This study found that fathers often felt left out of the decision making process and that their role was primarily one of providing supportive care to the mother [30]. However, fathers who were interviewed were conflicted as to how to provide support during specific breastfeeding challenges and were thus, more likely to support the mother’s decision to stop breastfeeding [30]. There is a growing body of literature suggesting that fathers should be included in both the breastfeeding decision making process and acquisition of positive, functional support behaviors postpartum [8,27,31-34]. Fathers will require information on the ways in which they can best support mothers in meeting breastfeeding challenges.

Timing of support is important in the initiation and maintenance of breastfeeding but also in the development of maternal confidence. Learning the skills of latching and positioning the baby early in the postpartum period is critical to establishing breastfeeding patterns and breast milk supply. Faced with challenges such as engorgement, latching difficulties, fatigue and perceived insufficient milk production, women without support and resources are likely to “give up” [6,7,10]. Partners who are present during this period could offer timely support and encouragement. Armed with information and rudimentary skills they could be engaged in targeted support activities. Timely partner intervention may be crucial to the continuance of breastfeeding.

Our results indicate that active support measures such as preparing baby and bringing beverages coupled with positive verbal phrases encouraged and sustained maternal confidence in breastfeeding. Health care professionals providing assistance in the prenatal and postpartum periods have an opportunity to help partners recognize that breastfeeding ‘is best for baby’ as well as the effect of encouraging words and deeds on mothers’ confidence and decision to continue breastfeeding. Strategies to actively support breastfeeding have a greater impact on sustaining mothers’ efforts. Verbal and nonverbal encouragement to mothers from fathers was reported by Rempel and Rempel to facilitate breastfeeding and was more likely to occur when fathers had increased knowledge about breastfeeding and used it to assist with breastfeeding challenges [33].

Involving fathers in breastfeeding will require increased efforts on the part of health care professionals to dispel the “exclusivity” of the mother/baby dyad. One of these efforts will be to offer information to partners so they can formulate knowledgeable solutions given problems they may witness. In 2006, Pisacane et al. found that teaching fathers (intervention group) about “fear of milk insufficiency, transitional lactation crisis, return to outside employment, and problems such as breast engorgement, mastitis, sore and inverted nipples, and breast refusal” and preventive and management techniques resulted in significant differences in successful lactation and increasing breastfeeding rates compared to the control group [11]. It should be noted that not all partners may be interested in this level of involvement. However, our results indicated that supportive partner interaction, advice on how to be patient and to persevere throughout breastfeeding challenges was the most useful for women.

We found that responses to the HHLS were indistinguishable between our two groups termed active/positive and ambivalent/negative. As one of the sub-scales measured maternal confidence and commitment, we would have expected that maternal perception of active/positive support from fathers may have positively affected maternal confidence and this could have differentiated the groups. It could be that the confidence in the ability to breastfeed as measured by the BSES is more affected by partner support than maternal perception of milk production or the maternal assessment of infant satiety.

Key breastfeeding supports were identified by participants. Interestingly, for our sample, health care professionals and mother/friends ranked almost equally but we note that their information bases may be different. Health care professionals offer evidence-based information whereas mothers/friends may have their information grounded in experience, folk lore, and television, internet or media sources. Prenatal class information was also identified as being helpful therefore relevant and accurate breastfeeding content should be maintained.

Our research focused on maternal perception of breastfeeding support not on the actions of partners. Consequently, we cannot conclude that partners were not providing instrumental, emotional, or other forms of support, only maternal perception of that support. Information could be provided to fathers to attend to how mothers wish to be supported during the prenatal and postpartum period and give examples of active and positive supportive behaviors.

### Limitations

We used a convenience sample which is subject to selection bias and threatens the internal validity of our study. Generalizability is limited by the small sample size of our findings to other breastfeeding women. Another limitation is that women reported their perceptions of support at different times throughout the postpartum period thus a woman’s initial perception of partner support may have changed depending whether breastfeeding was maintained or stopped. Self-report is subject to recall bias and some women may have forgotten their initial perceptions of partner support. Verification of emergent categories with women would have validated the results.

## Conclusion

Mothers reporting positive support from their partners had higher confidence in breast milk production and higher breastfeeding self-efficacy as measured by the BSES. Our results concur with previous studies that partners can influence a woman’s confidence in breastfeeding [11,28,32]. More specifically, those fathers who actively support and encourage women by helping position the baby and bringing snacks and diapering, are highly influential in a mother’s perceptions of confidence in breastfeeding. It is important that health care professionals appreciate the influence positive and active partner support has upon maternal feelings of confidence in breastfeeding and offer partners tips on common support strategies. In both prenatal classes and during postpartum stay in hospital as well as postpartum visits, health professionals can invite partners to become active learners and highlight supportive functions that are known to be meaningful to mothers. For some, learning solutions to common breastfeeding challenges may offer an opportunity for mothers to have an in-house resource. Future research could address knowledge gaps partners may have, the effect of breastfeeding on sexual intimacy for both parents and explore the disconnectedness partners have reported.

## Competing interests

The authors declare that they have no competing interests.

## Authors’ contributions

CAM developed the design for the study and drafted the manuscript. AH made substantial contribution to the acquisition of data and was involved in manuscript revision. CAM, AH, SM and ST analyzed and interpreted the data and were involved in manuscript revision. All authors read and approved the final version.

## Authors’ information

CAM is an Associate Professor with the Faculty of Nursing, University of Calgary. AH is a former student with the Faculty of Nursing, University of Calgary. SM is a post doctoral fellow at the Child Development Centre, Alberta Children’s Hospital. ST is a Professor, Departments of Pediatrics and Community Health Sciences, Faculty of Medicine, University of Calgary Health Scholar, Alberta Heritage Foundation for Medical Research Scientific Director, Child Development Centre Alberta Children’s Hospital.
